# Editorial: Advanced echocardiographic techniques in structural heart intervention

**DOI:** 10.3389/fcvm.2022.970515

**Published:** 2022-07-18

**Authors:** Randolph H. L. Wong, Yiting Fan, Masaki Izumo, Alex P. W. Lee

**Affiliations:** ^1^Division of Cardiothoracic Surgery, Department of Surgery, The Chinese University of Hong Kong, Shatin, Hong Kong SAR, China; ^2^Division of Cardiology, Shanghai Jiao Tong University, Shanghai, China; ^3^Division of Cardiology, Department of Internal Medicine, St. Marianna University School of Medicine, Kawasaki, Japan; ^4^Division of Cardiology, Department of Medicine, The Chinese University of Hong Kong, Shatin, Hong Kong SAR, China

**Keywords:** Transesophageal Echocardiography, Transcatheter Aortic Valve Replacement, paravalvular leak, atrial septal defect occlude, Pulmonary Valve Stenosis, tricuspid valve intervention, Hypertrophic Obstructive Cardiomyopathy

“The real voyage of discovery consists not in seeking new landscapes, but in having new eyes”

-Marcet Proust (1871–1922), French Novelist

From the traditional methods of physical examination by palpation and auscultation, to invasive cardiovascular imaging with irradiation hazard and risk of contrast nephropathy, and to the current state-of-the-art 3-Dimensional echocardiography, clinicians have all long been trying to understand human pathophysiology through “new eyes.” This nicely echoed with what Marcet Proust mentioned over 100 years ago. With these “new eyes,” our horizon of non-invasive imaging is broadened and covering areas that we have not dreamed of. Advances in echocardiographic technologies have become our “new eyes” for various structural heart disease intervention including transcatheter left atrial appendage occlusion, valvular repair or replacement, as well as endovascular aortic interventions (1–5).

This timely Research Topic on “Advanced Echocardiographic Techniques in Structural Heart Intervention” features the collection of articles published in Frontiers in Cardiovascular Medicine which underscores the important role of advanced echocardiographic imaging in the modern structural cardiovascular interventions ranging from aortic valve interventions, treatment of paravalvular leak (PVL), tricuspid valve intervention, and to the catheter-based treatment of congenital heart diseases.

*Peri-procedural Trans-esophageal Echocardiographic Sizing of the Native Left Ventricular Outflow Tract During Edwards INTUITY Valve Implantation*, by Lim et al. Rapid deployment aortic valve replacement has been increasingly adopted in the surgical world. Like Trans-catheter Valve Replacement (TAVR), the rapid deployment valve faced the problem of having a higher rate of post-operative conduction disturbance and the need for permanent pacing maker. Lim et al., described a retrospective study and derived a novel way of Trans-esophageal Echocardiographic (TEE) measurement of the diameter of the native left ventricular outflow tract (LVOT) at the landing site of the sub-annular stent and found over-sizing the native LVOT by 5 mm or more was associated with an increased risk of new-onset conduction disturbances. The authors stressed the importance of incorporating TEE LVOT measurement to guide the choice of valve size to prevent post-operative conduction disturbance.

*Case Report: Minimally Invasive Therapy by Transcatheter Aortic Valve Replacement and Percutaneous Intramyocardial Septal Radiofrequency Ablation for a Patient With Aortic Stenosis Combined With Hypertrophic Obstructive Cardiomyopathy: Two-Year Follow-Up Results*, by Li et al. The authors presented a case of severe aortic valve stenosis with septal hypertrophy who was successfully treated with TAVR and percutaneous intramyocardial septal radiofrequency ablation (PIMSRA) under transthoracic echocardiography (TTE) guidance. The TTE evaluation at 2 years showed satisfactory gradient and septal thickness with evidence of relapse.

*Transcatheter Closure of a Paravalvular Leak Guided by Transesophageal Echocardiography and Three-Dimensional Printing*, by Xu et al. Paravalvular leak (PVL) after surgical valve replacement is a daunting complication with an incident of around 0.75–2.3% (6). Conventional treatment for symptomatic cases required re-operative open heart surgery and was associated with significant morbidities and mortality. Xu et al. reported their 166 patients with post-surgical valve replacement paravalvular leak and they compared patients' demographics, catheter procedural details and post-operative outcomes in those with TEE and 3D printing guidance (TEE&3D) and Transthoracic Echocardiographic (TTE) guidance. They found that the TEE&3D approach resulted in statistically significant shorter procedural and fluoroscopic time. Their study nicely highlighted how 3D printing technology assisted the correct choice of the percutaneous delivery system, and choice of occluder devices. It also helps biomedical engineers understand the anatomy of PVL and potentially facilitates the design and development of next-generation of dedicated devices for PVL closure.

*Transcatheter Tricuspid Valve Therapy: From Anatomy to Intervention*, by Cammellari et al. provides the readers a comprehensive review on the tailored imaging modalities, detailed anatomical account of tricuspid valve (TV) and its surrounding structures, as well as different catheter-based interventional procedures to treat patients with severe symptomatic tricuspid regurgitation (TR). This review serves as an all-you-need-to-know article to catch up with the state-of-the-art interventions for TV pathologies with succinctly constructed tables and nicely presented figures for optimal views from different imaging modalities such as 3D TEE and cardiac computered-tomography. Cardiac interventionists who are involved in TV edge-to-edge repair, tricuspid annuloplasty, caval implants, spacer, and catheter-based valve implantation will find this article to be very useful.

*Simultaneous Percutaneous Interventional Treatment of Atrial Septal Defects and Pulmonary Valve Stenosis in Children Under the Guidance of Transesophageal Echocardiography Alone: Preliminary Experiences*, by Lu et al. The authors described a series of irradiation-free, pure TEE guided atrial septal defect (ASD) closure and pulmonary valve (PV) balloon dilatation in children with ASD and PV stenosis which demonstrated the beauty of the advances in modern structural heart intervention that spared the patients from open-heart surgeries under cardiopulmonary bypasses. Technical procedural steps were described in detail including the rationale of the sequence of performing PV ballooning prior to ASD closure, saline contrast guided location of the catheter tip and sizing of the ASD device. Not only the patients were spared from the open-heart operation and irradiation hazards, all of them also showed tremendous improvements in pulmonary transvalvular gradient in subsequent follow-up.

*A Prospective, Single-Center, Phase I Clinical Trial to Evaluate the Value of Transesophageal Echocardiography in the Closure of Patent Foramen Ovale With a Novel Biodegradable Occluder*, by Du et al. Metal alloy occluders are the most used devices to close patent foramen ovale (PFO). However, such a device might limit the future transeptal access to the left atrium and left-sided heart valve diseases. Du et al. described a prospective, single center clinical study of 44 patients who underwent Novel Biodegradable Occluder (NBO) implantation under TTE guidance. Their subsequent follow-up at 3 months, 6 months and 1 year with TTE and TEE evaluation after the procedure confirmed PFO occlusion rate reached 95.5%. This concept of utilizing NBO will change the landscape of atrial septal treatment as it allows a future transeptal approach to the left atrial appendage and mitral valve.

## Author contributions

RW, YF, MI, and AL are topic editors of this Research Topic and contributed to the writing and revising of this editorial. All authors contributed to the article and approved the submitted version.

## Conflict of interest

The authors declare that the research was conducted in the absence of any commercial or financial relationships that could be construed as a potential conflict of interest.

## Publisher's note

All claims expressed in this article are solely those of the authors and do not necessarily represent those of their affiliated organizations, or those of the publisher, the editors and the reviewers. Any product that may be evaluated in this article, or claim that may be made by its manufacturer, is not guaranteed or endorsed by the publisher.
